# Association of Unhealthy Lifestyles with Cataract Risk, and The Mediating Role of Metabolic Signature: Analysis of the UK Biobank Prospective Cohort

**DOI:** 10.14336/AD.2025.0522

**Published:** 2026-07-23

**Authors:** Jiao Qi, Kaimeng Su, Keke Zhang, Wenwen He, Jiaqi Meng, Yu Du, Yi Lu, Xiangjia Zhu

**Affiliations:** ^1^Eye Institute and Department of Ophthalmology, Eye & ENT Hospital, Fudan University, Shanghai, 200031, China.; ^2^NHC Key Laboratory of Myopia and Related Eye Diseases, Key Laboratory of Myopia and Related Eye Diseases, Chinese Academy of Medical Sciences, Shanghai, 200031, China.; ^3^Shanghai Key Laboratory of Visual Impairment and Restoration, Shanghai, 200031, China.; ^4^State Key Laboratory of Medical Neurobiology, Fudan University, Shanghai, 200032, China

**Keywords:** Cataract, Unhealthy lifestyle, Metabolic signature, Mediation analysis

## Abstract

Emerging evidence has shown an association between certain unhealthy lifestyle factors and cataract risk. However, the synergistic effect of unhealthy lifestyle factors on cataract risk and their underlying mechanisms remains unknown. This study analyzed data from 199,415 baseline cataract-free participants from the UK Biobank prospective cohort study. Multivariable Cox proportional hazards models estimated the associations of individual unhealthy lifestyle factors (smoking, alcohol consumption, physical inactivity, unhealthy diet, and high body mass index) and their synergistic effect with cataract risk. Elastic net regression was performed to identify a metabolic signature reflecting unhealthy lifestyles, and the mediation effect was evaluated. Our results showed that only smoking and physical inactivity significantly increased cataract risk. Compared to the favorable lifestyle group, the cataract risk increased by 6% in the intermediate lifestyle group (95% confidence interval [CI]: 1.02-1.09) and by 14% in the unfavorable lifestyle group (95% CI: 1.08-1.20). The Cox regression model also revealed that the metabolic signature of unhealthy lifestyles was associated with cataract risk (adjusted hazard ratio, 1.31; 95% CI: 1.18-1.45). Mediation analysis demonstrated that the metabolic signature mediated the association between unhealthy lifestyles and cataract risk, with a mediation proportion of 18.01% (95% CI: 8.57-34.70%). Metabolic pathway analysis revealed that two metabolic pathways (fatty acids and lipoprotein particle concentration and size) played a crucial mediating role. Our study underscores novel insights into the effect of unhealthy lifestyle factors on cataract risk and the mediating role of metabolic factors in this process.

## INTRODUCTION

Cataracts are the most common eye disease and a major cause of global blindness among the older adult population [1, 2]. In 2020, approximately 100 million people worldwide aged over 50 years experienced blindness or moderate-to-severe visual impairment due to cataracts [3]. Cataracts significantly diminish visual health and quality of life in older adults, imposing escalating socioeconomic burdens on global healthcare systems [4, 5]. Therefore, cataract prevention has long been a leading public health objective and has attracted extensive attention globally.

As an age-related eye disease, the incidence of cataracts in older adults is closely related to acquired environmental factors [6]. There has been significant public concern regarding how to modulate these factors to prevent the onset and progression of cataracts. Among these, lifestyle factors play an important part. Previous studies have shown that many unhealthy lifestyle behaviors, including smoking [7, 8], alcohol consumption [7, 9], physical inactivity [7, 10], and poor dietary habits [7, 11], can lead to functional disorders and age-related systemic diseases. However, these studies were predominantly fragmented and primarily focused on associations between individual unhealthy lifestyle factors and cataract risk. For instance, independent investigations respectively showed that cataract risks were likely associated with smoking [12], alcohol consumption [13], physical inactivity [14, 15], diet [16, 17], and obesity [18]. Despite these findings, validation through large-scale prospective cohort studies has been lacking. More importantly, no research has systematically identified the combined effects of these unhealthy lifestyle factors on cataract risk, nor the detailed mechanisms linking this relationship.

The combined effects of unhealthy lifestyle factors on cataract risk are likely mediated by metabolic mechanisms. Established evidence indicates that unhealthy lifestyle factors such as smoking and physical inactivity modify blood metabolism. Specifically, altered amino acid profiles were correlated with smoking-induced cellular damage [19], while dysregulated lipid profiles and inflammatory pathways were linked to sedentary behavior [20, 21]. Research has demonstrated that these metabolites, particularly lipid-related metabolites, mediate the association between unhealthy lifestyles and the pathogenesis of chronic diseases such as type 2 diabetes and cardiovascular disorders [22, 23]. Cataracts are also closely related to metabolic dysregulation. For instance, aberrant cholesterol synthesis might disrupt lens protein homeostasis, potentially contributing to cataract formation [24, 25]. Therefore, we postulated that metabolic signatures may mediate the association between unhealthy lifestyles and cataract risk. However, evidence elucidating this hypothetical association remains conspicuously absent in current cataract-related research.

The UK Biobank, a large-scale prospective cohort with detailed phenotypic, lifestyle, and metabolic data from approximately 500,000 participants, provides an opportunity to address these knowledge gaps. By leveraging its longitudinal design, we aimed to investigate the impact of a combination of unhealthy lifestyle factors on cataract risk and identify the mediating plasma metabolic signature involved in this process. Our findings aim to inform targeted interventions for vision preservation, emphasizing the importance of holistic lifestyle modifications and metabolic treatments for cataract prevention.

## METHODS

### Study design and participants

This study collected extensive health-related information from participants in the UK Biobank prospective cohort study, which recruited over 500,000 participants aged 37-73 years at baseline between 2006 and 2010. The Northwest Multi-Center Research Ethics Committee approved the UK Biobank study (11/NW/0382), and all participants provided written informed consent. Our exclusion criteria included a history of cataract at baseline, incomplete lifestyle and metabolic data, and missing data on covariates. Ultimately, data from 199,415 individuals were included in the analysis. The sample selection procedure is shown in Supplementary Figure 1.

### Unhealthy lifestyles assessment

Consistent with the World Health Organization recommendations, we selected five unhealthy lifestyle factors: smoking, alcohol consumption, physical inactivity, unhealthy diet, and unhealthy high body mass index (BMI). Each of these was assessed at baseline via a touchscreen questionnaire. For each lifestyle factor, we assigned a score of 0 for healthy status and 1 for unhealthy status. We then selected the unhealthy lifestyle factors that significantly associated with cataract risk, assigned them equal weights, and calculated the unhealthy lifestyle scores. These scores were categorized into favorable, intermediate, and unfavorable levels. The definitions for unhealthy levels in the various lifestyle factors are provided below. The assigned levels were based on the information given by the participants at baseline using the touchscreen questionnaire. The data fields used for exposure assessment are listed in Supplementary Table 1.

#### Smoking assessment

An unhealthy smoking level was assigned to participants who reported having ever smoked, were currently smoking, or had smoked at least 100 cigarettes over their lifetime [26].

#### Alcohol consumption assessment

An unhealthy alcohol consumption level was assigned to participants, according to the UK dietary guidelines, who reported consuming more than one alcoholic drink daily for females and two drinks for males [26].

#### Physical activity assessment

Physical inactivity was defined by any of the following criteria: engaging in vigorous activity for fewer than 75 minutes per week, moderate activity for fewer than 150 minutes per week, an equivalent combination of both, vigorous activity less than once a week, or moderate activity fewer than five days a week [27, 28]. Total physical activity time was calculated based on the reported duration and number of days per week, plus an additional 10 minutes. The equivalent combination was calculated based on the metabolic equivalence conversion between activity intensity and duration, where vigorous activity duration was considered equivalent to twice the duration of moderate activity [29].

#### Dietary intake assessment

The participants reported the intake frequency of a range of common food and drink items. We selected 12 data fields for this study: beef, lamb/mutton, pork, processed meat, oily fish, non-oily fish, fresh fruit, dried fruit, salad/raw vegetables, cooked vegetables, bread, and cereal.

These food items were categorized into seven new data fields: red meat (beef, lamb, and pork), processed meat, total fish (oily and non-oily), total vegetables (cooked and raw), total fruits (fresh and dried), whole grains, and refined grains [30]. Grains were categorized into whole and refined grains based on the predominant bread and cereal types consumed. Whole meal or whole grain bread, bran cereals, oatmeal, and muesli were defined as whole grains, while white bread, brown bread, other bread varieties, biscuits, and other cereal products were considered refined grains.

In order to better classify and compare the dietary intake, we used unified units to define baseline serving sizes for each food type, as previously described [30]. Detailed serving sizes and corresponding codes for the food items and groups are presented in Supplementary Table 1.

The cut-off values for the seven dietary factors were based on prior research and guidelines promoting cardiometabolic health [31, 32]. The healthy diet score was based on the following dietary elements, each given one point: at least four daily servings of total fruits; at least four daily servings of total vegetables; at least two weekly servings of total fish; no more than one weekly serving of processed meat; no more than 1.5 weekly servings of red meat; at least three daily servings of whole grains; and no more than 1.5 daily servings of refined grains. The UK Biobank dataset exhibited distributional characteristics similar to the above cited study [31, 32], so we employed an identical scoring method. The score ranged from 0 to 7 points, with an unhealthy diet considered when the score was below 4 points.

#### BMI assessment

The BMI was calculated based on measurement data collected during the initial assessment visit. Unhealthy BMI was defined as a BMI outside the range of 18.5-24.9 kg/m^2^.

### Ascertainment of cataract and follow-up time

Our study focused on incident cataracts as the exposure outcome [33, 34]. Incident cataracts were determined based on three criteria: (1) ICD-10 codes H25, H250, H251, H252, H258, and H259, and ICD-9 code 3661; (2) self-reported medical condition code 1278 in UK Biobank data field 20002; and (3) participant reporting of cataract in the touchscreen questionnaire's eye issues/disorders item (data field 6148). The age at cataract diagnosis, as reported in data field 4700, was also noted [35].

The follow-up time was calculated from the baseline assessment until the earliest of the following events: date of incident cataract, death, loss to follow-up, or administrative censoring as the end of follow-up (October 31, 2022 for Hospital Episode Statistics for England, July 31, 2021 for Scottish Morbidity Record for Scotland, and February 28, 2018 for Patient Episode Database for Wales).

### Identification of the metabolic signature reflecting unhealthy lifestyles

Metabolic data were derived from EDTA plasma samples of 280,000 UK Biobank participants. The high-throughput NMR-based metabolomic biomarker analysis platform, developed by Nightingale Health Ltd., quantified 251 metabolic biomarkers. Measurements were conducted in two phases: June 2019 to April 2020, and April 2020 to June 2021. Only baseline samples from participants with over 80% of metabolites successfully measured were included in this study. Our analysis included 249 metabolites that passed quality control checks, comprising 168 metabolites with absolute levels (mmol/L) and 81 presented as ratios. These metabolites span multiple metabolic pathways, including 14 subclasses of lipoprotein lipids, fatty acid compositions, and various low-molecular-weight metabolites. The concentration values of the 249 metabolites were log-transformed (ln[x+1]) and then Z-transformed before analysis. Metabolites without data were imputed with a mean of 0.

We adopted an elastic net regression model to establish a metabolic signature reflecting the unhealthy lifestyle score. This model combines LASSO and Ridge penalties, reducing model overfitting and enhancing the result reliability [36]. Participants were divided into training and test sets in an 8:2 ratio. The ideal lambda parameter was determined via a 10-fold cross-validation process, selecting the largest value that yielded a mean squared error within one standard error of the minimum observed value. The metabolic signature was calculated by computing the linear combination of metabolites exhibiting non-zero coefficients, with their contributions scaled according to their respective coefficient values [37]. Subsequently, we performed pathway analysis for metabolites reflecting unhealthy lifestyles. This analysis was based on the detailed grouping of metabolic biomarkers in the Nightingale Heath-generated dataset to further explore the underlying mechanisms linking unhealthy lifestyles and cataract risk.

### Covariates

Several covariates were adjusted for in the analysis, including age, sex, ethnicity, the Townsend deprivation index (TDI), income, education, diabetes, and hypertension. Sociodemographic factors such as age, sex, ethnicity, income, and education scores were self-reported at baseline. Ethnicity was categorized as white and non-white. The TDI score, ranging from -6.26 to 10.82, was assigned based on the residential postal code, with a higher index score indicating a greater level of relative poverty within that area. Diabetes and hypertension were diagnosed based on medical documentation using ICD-10 codes E10-E14 and I10-I15, respectively.

### Statistical analysis

Baseline characteristics were summarized for five lifestyle factors. Categorical variables are expressed as frequencies (percentages), and continuous variables as means ± standard deviations (SDs). Cox proportional hazards models, including one unadjusted and two adjusted models, were used to estimate hazard ratios (HRs) and their 95% confidence intervals (CIs) for the associations of each unhealthy lifestyle factor, unhealthy lifestyle score, the metabolic signature, and individual metabolites with the risk of incident cataract. Unhealthy lifestyle scores were calculated using the individual unhealthy lifestyle factors that were significantly associated with cataract risk in preliminary analyses. For example, if smoking and unhealthy physical activity were the selected unhealthy lifestyle factors, the unhealthy lifestyle score was calculated based on the participants' unhealthy smoking and inactivity status. Accordingly, participants were categorized into favorable (none), intermediate (one), or unfavorable (both) subgroups. Model 1 was adjusted for age and sex. Model 2 was further adjusted for ethnicity, TDI, income, education, diabetes, and hypertension. A cumulative incidence curve was plotted to illustrate the 10-year incidence rate of cataract according to various levels of unhealthy lifestyles. Logistic regression was performed to estimate the associations between each metabolite in the unhealthy lifestyle's metabolic signature and each unhealthy lifestyle factor, after adjusting for potential confounders.

Mediation analysis, using the *mediation* package in R, investigated whether a metabolic signature mediated the relationship between unhealthy lifestyles and cataract risk. Briefly, this analysis aimed to estimate the proportional direct and indirect effects in the exposure-outcome relationship through mediators, considering the overall association. The mediator and incident cataract outcome models were adjusted for the same covariates as described above. The mediation analysis employed a quasi-Bayesian approach for variance estimation across 100 simulations. The mediated proportion was calculated as the ratio of the indirect effect to the total effect, with 95% CIs determined using the bootstrap method [38-40]. Additionally, the mediation effects of each metabolite and metabolic pathway within the metabolic signature associated with unhealthy lifestyles and cataract risk were examined.

Subgroup analysis was performed, stratified by age (<60 *vs.* ≥60 years), sex (male vs. female), and diabetes (with vs. without). Sensitivity analysis was conducted after excluding participants who developed cataracts within two years of baseline enrollment. Statistical analysis was performed using R Studio (version 4.4.2). A two-tailed *P* < 0.05 was considered statistically significant.

## RESULTS

### Baseline characteristics of the study participants

The baseline characteristics of the study participants, stratified by lifestyle factors, are presented in Table 1. The study included data from 199,415 UK Biobank participants, with a mean age of 56.1 ± 8.1 years, and 51.7% were female. Within the entire cohort, prevalent unhealthy lifestyle factors included smoking in 89,930 participants (45.1%), alcohol consumption in 192,068 participants (96.3%), physical inactivity in 37,974 participants (19.0%), unhealthy diet in 148,341 participants (74.4%), and unhealthy BMI in 135,226 participants (67.8%). During a median follow-up of 13.4 years, 16,908 (8.5%) incident cataracts were recorded. The incidence rates of cataract in the unhealthy lifestyle groups were higher than in their healthy counterparts for smoking (9.2% [8262/89930] vs. 7.9% [8646/109485]) and BMI (8.8% [11930/135226] vs. 7.8% [4978/64189]).

**Table 1:** Baseline characteristics of the study participants by lifestyle.

	Lifestyle									
	Smoking		Alcohol consumption		Activity		Diet		BMI	
	Healthy *n* = 109,485	Unhealthy *n* = 89,930	Healthy *n* = 7,347	Unhealthy *n* = 192,068	Healthy *n* = 161,441	Unhealthy *n* = 37,974	Healthy *n* = 51,074	Unhealthy *n* = 148,341	Healthy *n* = 64,189	Unhealthy *n* = 135,226
Incident cataract, *n* (%)	8646 (7.9)	8262 (9.2)	795 (10.8)	16113 (8.4)	13716 (8.5)	3192 (8.4)	4748 (9.3)	12160 (8.2)	4978 (7.8)	11930 (8.8)
**Sociodemographic characteristics**										
Age, years, mean ± SD	55.4±8.1	57.0 ± 8.0	56.6 ± 8.6	56.1 ± 8.1	56.2 ± 8.1	55.7 ± 7.8	57.0 ± 7.8	55.8 ± 8.2	55.3 ± 8.2	56.5 ± 8.0
Sex, *n* (%)										
Male	48044 (43.9)	48317 (53.7)	2250 (30.6)	94111 (49.0)	77795 (48.2)	18566 (48.9)	18353 (35.9)	78008 (52.6)	23409 (36.5)	72952 (53.9)
Female	61441 (56.1)	41613 (46.3)	5097 (69.4)	97957 (51.0)	83646 (51.8)	19408 (51.1)	32721 (64.1)	70333 (47.4)	40780 (63.5)	62274 (46.1)
Ethnicity, *n* (%)										
White	103484 (94.5)	86911 (96.6)	5407 (73.6)	184988 (96.3)	154291 (95.6)	36104 (95.1)	48545 (95.0)	141850 (95.6)	61452 (95.7)	128943 (95.4)
Non-white	6001 (5.5)	3019 (3.4)	1940 (26.4)	7080 (3.7)	7150 (4.4)	1870 (4.9)	2529 (5.0)	6491 (4.4)	2737 (4.3)	6283 (4.6)
Education, mean ± SD	12.86 ± 14.74	15.47 ± 16.90	18.01 ± 17.95	13.88 ± 15.70	13.69 ± 15.51	15.52 ± 16.93	12.80 ± 14.73	14.46 ± 16.14	11.93 ± 14.32	15.03 ± 16.37
Townsend deprivation index, mean ± SD	-1.80 ± 2.82	-1.18 ± 3.11	-0.60 ± 3.36	-1.56 ± 2.95	-1.58 ± 2.94	-1.28 ± 3.10	-1.58 ± 2.93	-1.58 ± 2.94	-1.69 ± 2.88	-1.44± 3.01
Income, mean ± SD	1.07 ± 4.70	1.32 ± 5.83	1.37 ± 5.93	1.17 ± 5.21	1.13 ± 5.12	1.39 ± 5.72	1.05 ± 4.87	1.23 ± 5.36	0.97 ± 4.46	1.28 ± 5.57
Hypertension, *n* (%)	38311 (35.0)	30192 (43.4)	3176 (43.2)	74162 (38.6)	60657 (37.6)	16681 (43.9)	18995 (37.2)	58343 (39.3)	15563 (24.2)	61775 (45.7)
Diabetes mellitus, *n* (%)	4332 (4.0)	5351 (6.0)	655 (8.9)	9028 (4.7)	6888 (4.3)	2795 (7.4)	2245 (4.4)	7438 (5.0)	1114 (1.7)	8569 (6.3)

Abbreviations: BMI, body mass index; SD, standard deviation.

### Impact of individual unhealthy lifestyle factors on cataract risk

We initially examined the relationship between individual unhealthy lifestyle factors and cataract risk (Table 2). After adjusting for potential confounding factors, we found that participants with unhealthy smoking habits (adjusted HR, 1.07; 95% CI: 1.03-1.10; *P* < 0.001) and physical inactivity (adjusted HR, 1.06; 95% CI: 1.02-1.10; *P* = 0.003) had a higher risk of developing cataract. The other three lifestyle factors (alcohol consumption, diet, and BMI) were not significantly associated with cataract risk.

**Table 2:** Associations between individual unhealthy lifestyle factors and cataract risk.

Lifestyle	Crude Model^a^		Model 1^b^		Model 2^c^	
	HR (95% CI)	*P*-Value	HR (95% CI)	*P*-Value	HR (95% CI)	*P*-Value
Smoking	1.20 (1.17-1.24)	<0.001	1.08 (1.05-1.12)	<0.001	1.07 (1.03-1.10)	<0.001
Drinking	0.76 (0.71-0.82)	<0.001	0.87 (0.81-0.94)	<0.001	0.95 (0.88-1.02)	0.166
Activity	1.00 (0.96-1.04)	0.921	1.10 (1.06-1.14)	<0.001	1.06 (1.02-1.10)	0.003
Diet	0.88 (0.85-0.91)	<0.001	1.01 (0.97-1.04)	0.671	1.00 (0.97-1.04)	0.799
BMI	1.16 (1.12-1.20)	<0.001	1.09 (1.05-1.12)	<0.001	1.01 (0.98-1.05)	0.513

a Unadjusted (crude) model.

b Adjusted for age and sex.

c Adjusted for age, sex, ethnicity, Townsend deprivation index (TDI), income, education, hypertension, and diabetes.

Abbreviations: HR, hazard ratio; CI, confidence interval.

### Impact of the unhealthy lifestyle score on cataract risk

The combined impact of the two influential unhealthy lifestyle factors (smoking and physical inactivity) on cataract risk is presented in Table 3. Participants were categorized into favorable (none), intermediate (anyone), and unfavorable (both) levels based on their unhealthy lifestyle scores. Multivariable-adjusted Cox regression analysis revealed that these unhealthy lifestyle scores were significantly associated with a higher cataract risk.

**Table 3:** Associations of unhealthy lifestyle scores and the metabolic signature with cataract risk.

Group		Crude Model^a^		Model 1^b^		Model 2^c^	
		HR (95% CI)	*P*-Value	HR (95% CI)	*P*-Value	HR (95% CI)	*P*-Value
Lifestyle	Favorable	Ref		Ref		Ref	
	Intermediate	1.14 (1.10-1.17)	<0.001	1.08 (1.04-1.11)	<0.001	1.06 (1.02-1.09)	<0.001
	Unfavorable	1.23 (1.16-1.29)	<0.001	1.20 (1.14-1.27)	<0.001	1.14 (1.08-1.20)	<0.001

Metabolic signature		2.02 (1.84-2.22)	<0.001	1.70 (1.53-1.87)	<0.001	1.31 (1.18-1.45)	<0.001

a Unadjusted (crude) model.

b Adjusted for age and sex.

c Adjusted for age, sex, ethnicity, Townsend deprivation index (TDI), income, education, hypertension, and diabetes.

Abbreviations: HR, hazard ratio; CI, confidence interval.

**Figure 1.**
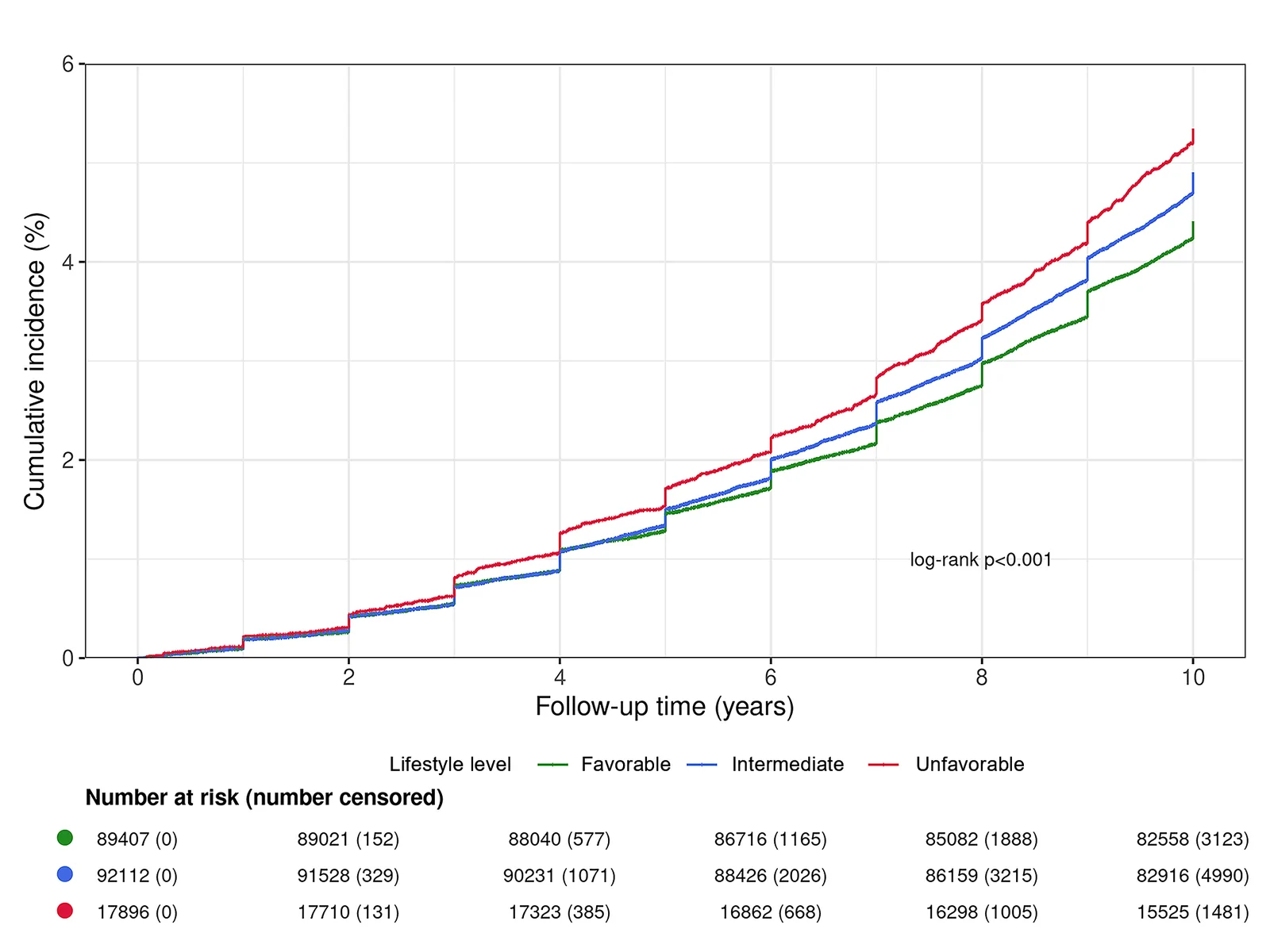
**Ten-year cumulative cataract incidence among UK Biobank participants by lifestyle level**. This Kaplan-Meier curve illustrates the cumulative cataract incidence among UK Biobank participants, categorized by lifestyle scores into 'Favorable' (green), 'Intermediate' (blue), and 'Unfavorable' (red) levels. The curve shows the number of participants at risk or censored over a ten year period, shown in two-year increments from baseline.

Compared to participants with a favorable lifestyle, those with an intermediate lifestyle showed a 6% higher risk of developing cataract (adjusted HR, 1.06; 95% CI: 1.02-1.09; *P* < 0.001), and those with an unfavorable lifestyle showed a 14% higher risk of developing cataract (adjusted HR, 1.14; 95% CI: 1.08-1.20; *P* < 0.001). These results demonstrate that the cumulative effect of multiple unhealthy lifestyle factors significantly increases cataract risk. Figure 1 presents the cumulative incidence curves of cataract by lifestyle levels. Ten years after the first follow-up, 8,827 participants developed cataracts. Compared with the favorable group (4.2% [3726/89407]), the cumulative cataract incidence was higher in the intermediate (4.6% [4206/92112]) and unfavorable (5.0% [890/17896]) groups (both *P* < 0.001), indicating that an increase in the total unhealthy lifestyle level was associated with a higher cumulative cataract incidence.

**Figure 2.**
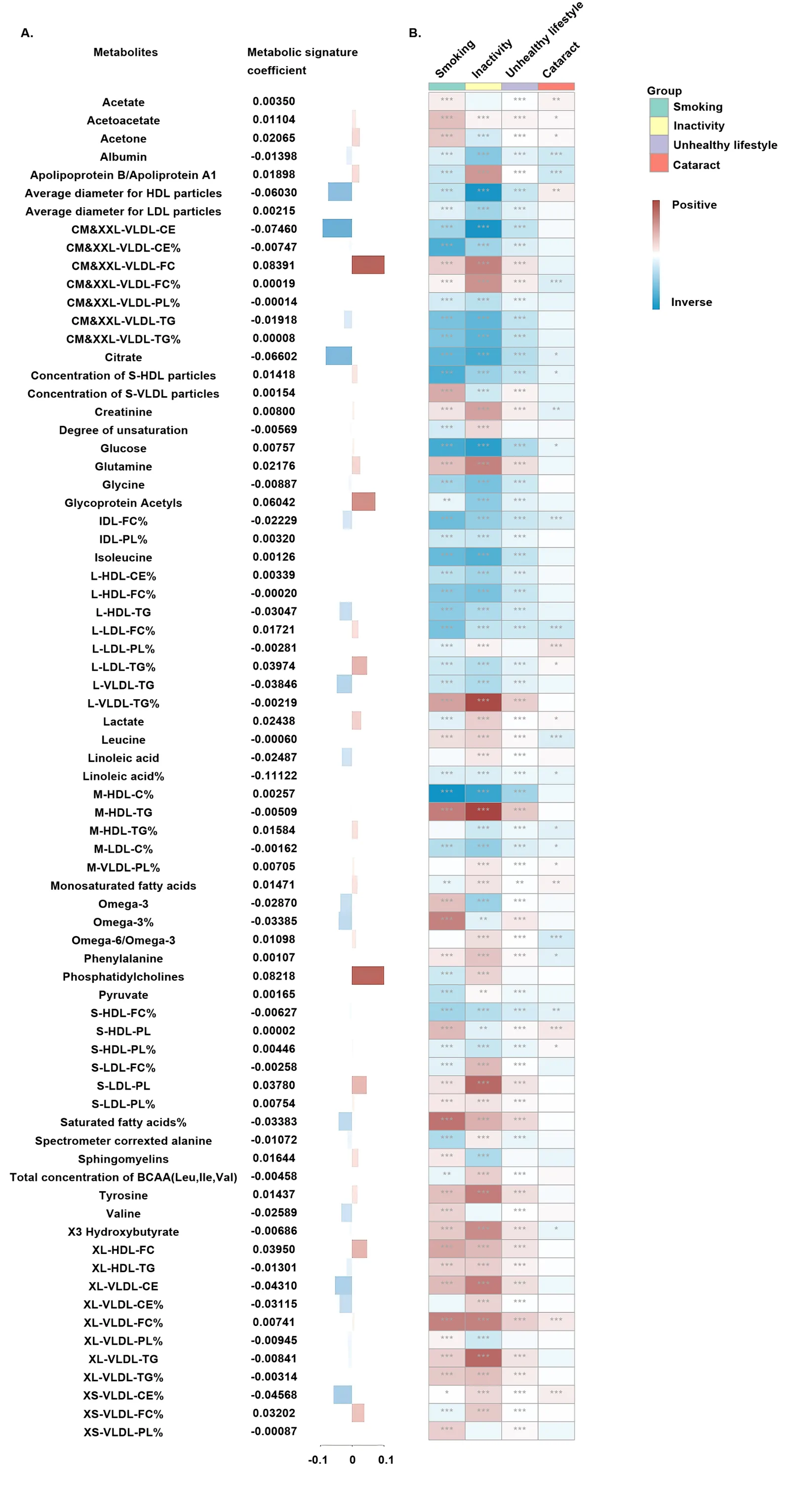
**Associations of metabolic signature metabolites with individual unhealthy lifestyle factors, unhealthy lifestyle scores, and cataract risk**. This figure presents (A) the coefficients of the 74 metabolites constituting the metabolic signature (bar chart), which indicate each metabolite's contribution to the metabolic signature, and (B) their associations with individual unhealthy lifestyle factors, unhealthy lifestyle scores, and cataract risk (heatmap), where the coefficients represent the natural logarithm of the hazard ratio [ln (HR)]. Specifically, HRs for associations between metabolites and unhealthy lifestyle factors (smoking and physical inactivity) reflect the relative metabolite changes per unit increase in that unhealthy lifestyle factor. Similarly, HRs for associations between metabolites and unhealthy lifestyle scores indicate the relative metabolite changes per unit increase in the score. The HR for cataract risk reflects the relative change in cataract risk per unit increase in the metabolites. The colors denote both the association directions (red = positive; blue = negative) and magnitudes (a darker color indicates a stronger magnitude). * *P* < 0.05, ** *P* < 0.01, *** *P* < 0.001). Abbreviations: C, cholesterol; CE, cholesteryl ester; FC, free cholesterol; CM, chylomicron; TG, triglyceride; BCAA, branched chain amino acid; Leu, leucine; Ile, isoleucine; Val, valine; PL, phospholipid; S, small; M, medium; VLDL, very LDL; XL, very large; XS, very small; XXL, extremely large.

**Figure 3.**
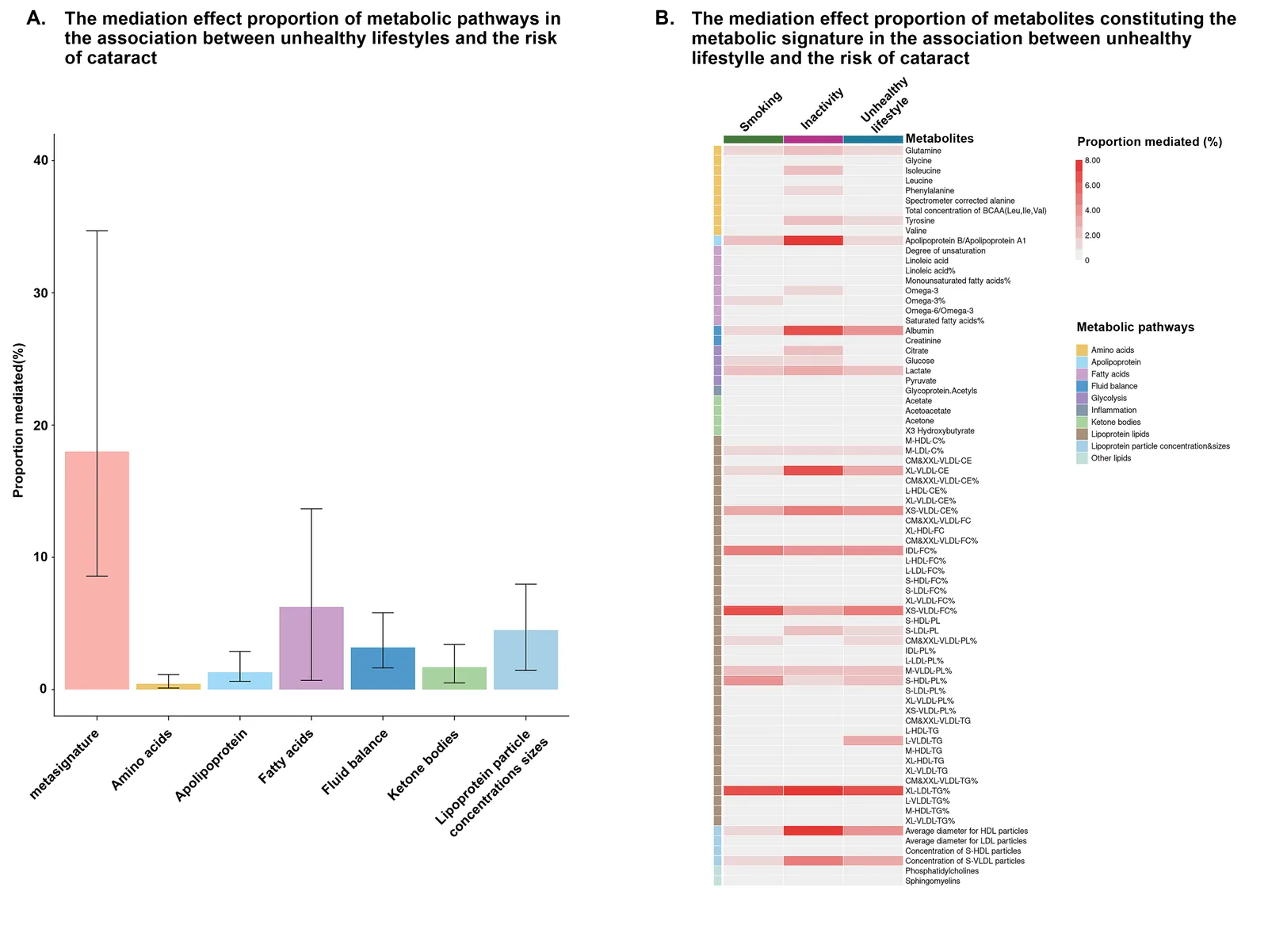
**Mediation proportions of metabolic pathways and metabolites in the association between unhealthy lifestyles and cataract risk**. (**A**) This panel illustrates the mediation proportion of metabolic pathways in the association between unhealthy lifestyles and cataract risk. Only statistically significant results (*P* < 0.05) are presented as bar charts. (**B**) This panel displays the mediation proportion of metabolites within the metabolic signature in the association between unhealthy lifestyles and cataract risk. The color for a metabolite's mediating effect is left blank if it lacks statistical significance (*P* > 0.05). Darker colors indicate stronger mediating effects for those with statistically significant mediation results. glyceride; BCAA, branched chain amino acid; Leu, leucine; Ile, isoleucine; Val, valine; PL, phospholipid; S, small; M, medium; VLDL, very LDL; XL, very large; XS, very small; XXL, extremely large; HR, hazard ratio; CI, confidence interval.

### Metabolic signature in response to unhealthy lifestyles

Elastic network regression identified a metabolic signature of unhealthy lifestyles, comprising 74 metabolites with non-zero coefficients (Fig. 2). These metabolites span various metabolic classes, including amino acids, apolipoproteins, fatty acids, fluid balance-related metabolites, glycolysis-related metabolites, inflammation-related metabolites, ketone bodies, lipids, and lipoproteins. The main contributors to the metabolic signature's positive coefficient were the ratio of free cholesterol (FC) to total lipids in chylomicrons and extremely large very low-density lipoproteins (XXL-VLDL), phosphatidylcholine, and glycoprotein acetyls. Conversely, the ratio of cholesteryl esters (CEs) to total lipids in chylomicrons and XXL-VLDL was inversely associated with the metabolic signature coefficient, contributing strongly to its negative direction.

### Impact of the metabolic signature on cataract risk

Multivariable-adjusted Cox regression analysis revealed that the metabolic signature of unhealthy lifestyles was positively associated with cataract risk (adjusted HR, 1.31; 95% CI: 1.18-1.45; *P* < 0.001; Table 3). The associations between individual metabolites in the metabolic signature and cataract risk are shown in Figure 2. The top five metabolites in the metabolic signature that were associated with cataract risk were citrate, the average high-density lipoprotein (HDL) particle diameter, the ratio of CEs to total lipids in very small VLDL (XS-VLDL), CEs in very large VLDL (XL-VLDL), and the ratio of triglycerides (TGs) to total lipids in large low-density lipoproteins (L-LDL).

### Mediating roles of the metabolic signature in the association between unhealthy lifestyles and cataract risk

Regarding the mediation effects, we found that the metabolic signature mediated 18.01% (95% CI: 8.57-34.70%, *P* < 0.001) of the association between unhealthy lifestyles and cataract risk. The mediation effect was primarily attributed to fatty acids (mediation proportion = 6.25%; 95% CI: 0.70-13.67%) and lipoprotein particle concentration and size (mediation proportion = 4.50%; 95% CI: 1.46-7.97%; Fig. 3A). The five metabolites with the highest mediation proportions were the ratio of TGs to total lipids in L-LDL, the ratio of FC to total lipids in XS-VLDL and intermediate density lipoprotein (IDL), the ratio of CEs to total lipids in XS-VLDL, and albumin (Fig. 3B).

### Subgroup and sensitivity analyses

Subgroup analyses, stratified by age, sex, and diabetes, are presented in Supplementary Figure 2. No statistically significant association was observed between unhealthy lifestyles and cataract risk among participants under 60 years. However, a significant association was identified in those aged ≥60 years, showing elevated risk in the intermediate (adjusted HR, 1.09; 95% CI: 1.05-1.13; *P* < 0.001) and unfavorable (adjusted HR, 1.16; 95% CI: 1.08-1.24; *P* < 0.001) lifestyle groups. Sex-specific analyses revealed that unhealthy lifestyles were associated with cataract risk in both males and females, but males showed stronger susceptibility (intermediate lifestyle: HR, 1.04; 95% CI: 1.00-1.09 for females vs. HR, 1.08; 95% CI: 1.02-1.13 for males; unfavorable lifestyle: HR, 1.11; 95% CI: 1.03-1.20 for females vs. HR, 1.18; 95% CI: 1.09-1.27 for males). Unhealthy lifestyle was not significantly associated with cataract risk among individuals with diabetes, but it was significantly associated among those without diabetes in both the intermediate (HR, 1.06; 95% CI: 1.03-1.10) and unfavorable (HR, 1.15; 95% CI: 1.09-1.22) lifestyle groups. These findings suggest that cataract pathogenesis in younger individuals (<60 years) or patients with diabetes may predominantly stem from non-lifestyle factors. Further analysis of the relationship between the metabolic signature and cataract risk across subgroups (Supplementary Table 2) revealed that the metabolic signature was significantly associated with cataract risk in all subgroups except for individuals with diabetes. The mediating role of the metabolic signature in the association between unhealthy lifestyles and cataract risk was exclusively identified in the ≥60 age group (mediation proportion, 11.03%; 95% CI: 2.63-22.62%; *P* < 0.001; Supplementary Table 3), males (mediation proportion, 10.77%; 95% CI: 0.90-37.30%; *P* < 0.001; Supplementary Table 3), and the non-diabetic population (mediation proportion, 16.86%; 95% CI: 5.80-32.35%; *P* < 0.001; Supplementary Table 3).

We performed a sensitivity analysis by excluding participants who developed cataracts within two years of recruitment to test the robustness of the results. The analysis showed that both unhealthy lifestyle and the metabolic signature were significantly associated with cataract risk (Supplementary Table 4). The metabolic signature of unhealthy lifestyle showed a mediating role in the association between unhealthy lifestyles and cataract risk (mediation proportion, 18.94%; 95% CI: 9.83-43.02%; *P* < 0.001). As in the entire cohort, the mediation effects in sensitivity analysis were primarily attributed to fatty acids (mediation proportion = 6.50%; 95% CI: 1.86-16.33%, *P* = 0.02, Supplementary Fig. 3), and lipoprotein particle concentration and size (mediation proportion = 3.94%; 95% CI: 1.84-8.57%, *P* < 0.001, Supplementary Fig. 3).

## DISCUSSION

Cataracts are a leading cause of blindness among older adults worldwide [1, 2]. Recent studies have found that some unhealthy lifestyle factors are associated with cataract risk [41, 42]; however, prior investigations had several limitations. These include a lack of systematic evaluation of unhealthy lifestyles, insufficient validations in large-scale prospective cohorts, and an oversight of potential metabolic involvement in this lifestyle-cataract relationship. This large prospective cohort study utilized data from the UK Biobank to demonstrate that unhealthy lifestyle factors, including smoking and physical inactivity, both individually and in combination, were associated with cataract risk. Furthermore, we showed that a metabolic signature mediated this association.

Among the unhealthy lifestyle factors assessed, smoking and physical inactivity were associated with an increased cataract risk. Consistent with previous studies [43], we identified smoking as an independent environmental risk factor for cataracts. Chemical components from smoke induce oxidative damage to the lens, and nicotine might trigger ocular vasoconstriction, contributing to cataract formation [43]. Existing evidence suggests that physical activity may prevent cataracts through multiple pathways, including antioxidation, anti-inflammation, and improved glucose and lipid metabolism [14]. These earlier findings indirectly highlight potential mechanisms by which physical inactivity could exacerbate cataract risk. Our study further revealed that exposure to multiple unhealthy lifestyles synergistically increased cataract risk, underscoring that lifestyle modifications, specifically regarding smoking and physical inactivity, may effectively reduce the cataract risk.

We successfully established a metabolic signature that reflects unhealthy lifestyles. This signature revealed that unhealthy lifestyles can significantly alter the body's metabolism, specifically by altering the ratio of FC to total lipids in chylomicrons and XL-VLDL, phosphatidyl-choline, glycoprotein acetyls, and other components. We further discovered that most metabolites linked to unhealthy lifestyles were also significantly associated with cataract formation, suggesting that metabolic changes induced by unhealthy lifestyles may contribute to the pathogenesis and progression of cataracts. Therefore, we used mediation analysis to identify the metabolic signature's mediating role in the association between unhealthy lifestyles and cataract risk, thereby proving the involvement of metabolic mechanisms in mediating this process.

We found that the involved metabolic mechanisms were primarily associated with fatty acids and lipoprotein particle concentration and size. Previous metabolomic analyses identified differentially expressed metabolites predominantly associated with the unsaturated fatty acid biosynthesis pathway in the aqueous humor of patients with cataracts [44]. Unhealthy lifestyles, such as smoking and physical inactivity, elevated circulating free fatty acids (FFAs) [10, 45-47], which aligns with our finding regarding the mediation effect of fatty acids. As albumin is the primary binding carrier for FFAs [48], excessive FFA concentrations might result in an increased molar ratio of FFA to albumin and could saturate binding sites. This saturation, in turn, might trigger mitochondrial dysfunction and damage lens epithelial cells, potentially contributing to cataract formation [49]. Regarding lipoprotein particle concentration and sizes, studies have demonstrated that a decrease in serum HDL cholesterol levels is an independent risk factor for cataracts [50], whereas oxidized LDL might promote cataracts by inducing transcriptomic abnormalities in lens epithelial cells [51].

Several relative lipoproteins to lipid concentrations were identified as critical mediators, including the ratios of TGs to total lipids in L-LDL, FC to total lipids in XS-VLDL and IDL, CEs to total lipids in XS-VLDL, and the albumin level. Our core findings are consistent with previous research [10, 44-51], indicating that disordered lipid metabolism is a critical metabolic mechanism in cataract pathogenesis. However, a key distinction lies in the focus: prior studies primarily focused on lens cell metabolism, while our research focused on systemic circulation levels. For example, significant disturbances in glycolipid and glycosphingolipid metabolism and significant dysregulation of the glycerophospholipid metabolic pathway in lens epithelial cells have been strongly associated with cataract formation [52, 53]. In contrast, our research revealed that alterations in the proportions of specific lipoproteins were associated with cataract risk. These alterations may represent upstream or related factors that either cause or accompany the intracellular lipid metabolic disorders observed in previous studies. Regarding albumin as a key mediator, prior research documented an imbalance in albumin redox status in patients with cataracts, characterized by lower reduced albumin and higher oxidized albumin levels in the serum and aqueous humor, which aligns with the findings of the present study [54, 55].

Beyond our study, which assessed metabolomic changes in patients with cataracts through a large-scale cohort, a recent metabolomic study of 192 patients with cataracts reported elevated carnitine levels and altered taurine levels in their lens. These findings suggest disruptions in fatty acid metabolism and antioxidant defense. This aligns well with our findings that fatty acid metabolism is a key metabolic mechanism linking unhealthy lifestyles to cataract development [56].

In subgroup analyses, we observed no statistically significant association between unhealthy lifestyles and cataract risk among participants under the age of 60 years. This may be attributed to the fact that non-lifestyle-related factors, such as trauma, electric shock, glucocorticoid usage, and ocular surgery, likely influenced cataract risk in this younger subgroup [57-61]. Notably, neither unhealthy lifestyle patterns nor related metabolic profiles demonstrated a statistically significant association with cataract risk among participants with diabetes. This discrepancy might stem from distinct mechanistic pathways. In patients with diabetes, cataracts might arise from abnormal accumulation of glucose and its metabolites, including sorbitol, whereas in non-diabetic participants [62], cataract development associated with unhealthy lifestyles appears mechanistically divergent.

Building on this study, we plan to expand our research into both fundamental mechanisms and clinical translation. For fundamental research, we will focus on causally validating key metabolites by investigating their effects on mitochondrial function, oxidative stress, and lipid deposition in lens epithelial cells to elucidate direct pathogenic mechanisms. For clinical translation, we plan to leverage metabolomic biomarkers that were confirmed to play a clear mediating role through our current study and future foundational experiments. Using statistical modeling, machine learning, and other methods, we will integrate core metabolites with unhealthy lifestyle factors to construct a preliminary prediction model. If this model achieves high predictive accuracy in future training and validation cohorts, it could function as a robust cataract risk prediction model, enabling early screening and targeted interventions.

Our study has systematically addressed the metabolic mechanisms underlying the association between unhealthy lifestyles and cataract risk. Nevertheless, certain limitations need to be noted. Firstly, while this study primarily focused on metabolomics analysis, subsequent studies could integrate genomics and proteomics to conduct multi-omics analyses, expanding the exploration of associations and mechanisms between unhealthy lifestyles and cataract risk. Secondly, due to limitations in the UK Biobank database, specifically the absence of diagnostic details on cataract subtypes (such as age-related, highly myopic, and diabetic cataracts), this study focused on total cataract cases without stratifying analyses by specific classifications. The clustering of all cataract subtypes represents a significant limitation, as the observed metabolic effects may reflect heterogeneous biological pathways between unhealthy lifestyles and different subtypes, potentially weakening our effect estimates. Thirdly, to explore the synergistic effect of several unhealthy lifestyle factors, we adopted a dichotomous classification. However, this approach might introduce bias into the effect estimates of unhealthy lifestyle exposure on the outcome variable. Moreover, due to the underrepresentation of diverse ethnicities among UK Biobank participants, most individuals analyzed in this study were White. Consequently, our findings may have limited applicability to other people. Additional limitations include potential unmeasured confounders (e.g., chronic stress, occupational exposures) affecting lifestyle, metabolism, and cataract risk, which could create spurious mediation pathways. Furthermore, reverse causation is possible, as early cataract symptoms might lead to lifestyle changes (e.g., reduced activity, altered BMI, smoking, and alcohol consumption). Future studies may utilize alternative data sources to conduct prospective studies focusing on distinct cataract subtypes, further elucidating the associations between unhealthy lifestyles and the risks of different cataract subtypes.

### Conclusions

In conclusion, this large-scale prospective cohort study demonstrated that unhealthy lifestyles, including smoking and physical inactivity, increase cataract risk. Furthermore, it showed that a metabolic signature played a mediating role in this association. Our results provide actionable guidance for cataract prevention through lifestyle modifications and advance mechanistic insights into its pathogenesis by identifying a mediating metabolomic signature.

## Supplementary Materials

The Supplementary data can be found online at: www.aginganddisease.org/EN/10.14336/AD.2025.0522.



## Data Availability

The datasets generated and analyzed, and the code applied during the current study, are available from the corresponding author upon reasonable request.
