# Supplementary material for: Association of Unhealthy Lifestyles with Cataract Risk, and The Mediating Role of Metabolic Signature: Analysis of the UK Biobank Prospective Cohort
Source: Aging Dis. 2026 Jul 23;17(5):2654–66. doi: 10.14336/AD.2025.0522 (PMC13437103; doi:10.14336/AD.2025.0522)
Supplement: Supplementary file 1 [file AD-17-5-2654-s.pdf]

SUPPLEMENTARY DATA

Association of Unhealthy Lifestyles with Cataract Risk, and The Mediating Role of Metabolic Signature: Analysis of the UK Biobank Prospective Cohort

Jiao Qi, Kaimeng Su, Keke Zhang, Wenwen He, Jiaqi Meng, Yu Du, Yi Lu, Xiangjia Zhu

© 2025. Qi J et al. Published online at http://www.aginganddisease.org/EN/10.14336/AD.2025.0522

SUPPLEMENTARY DATA

Supplementary Figure 1. The whole procedure of study sample selection

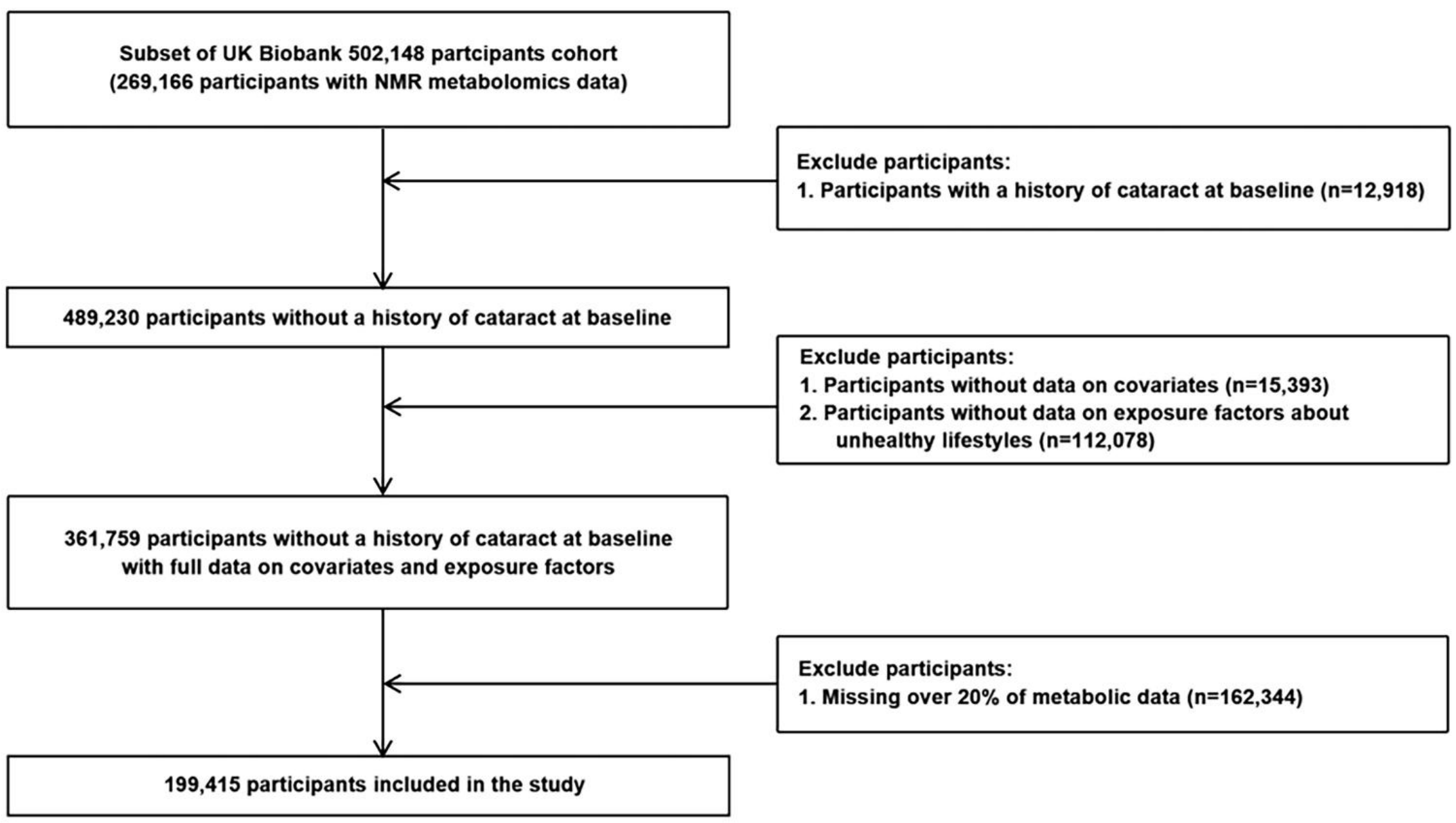

Supplementary Figure 1. The whole procedure of study sample selection.

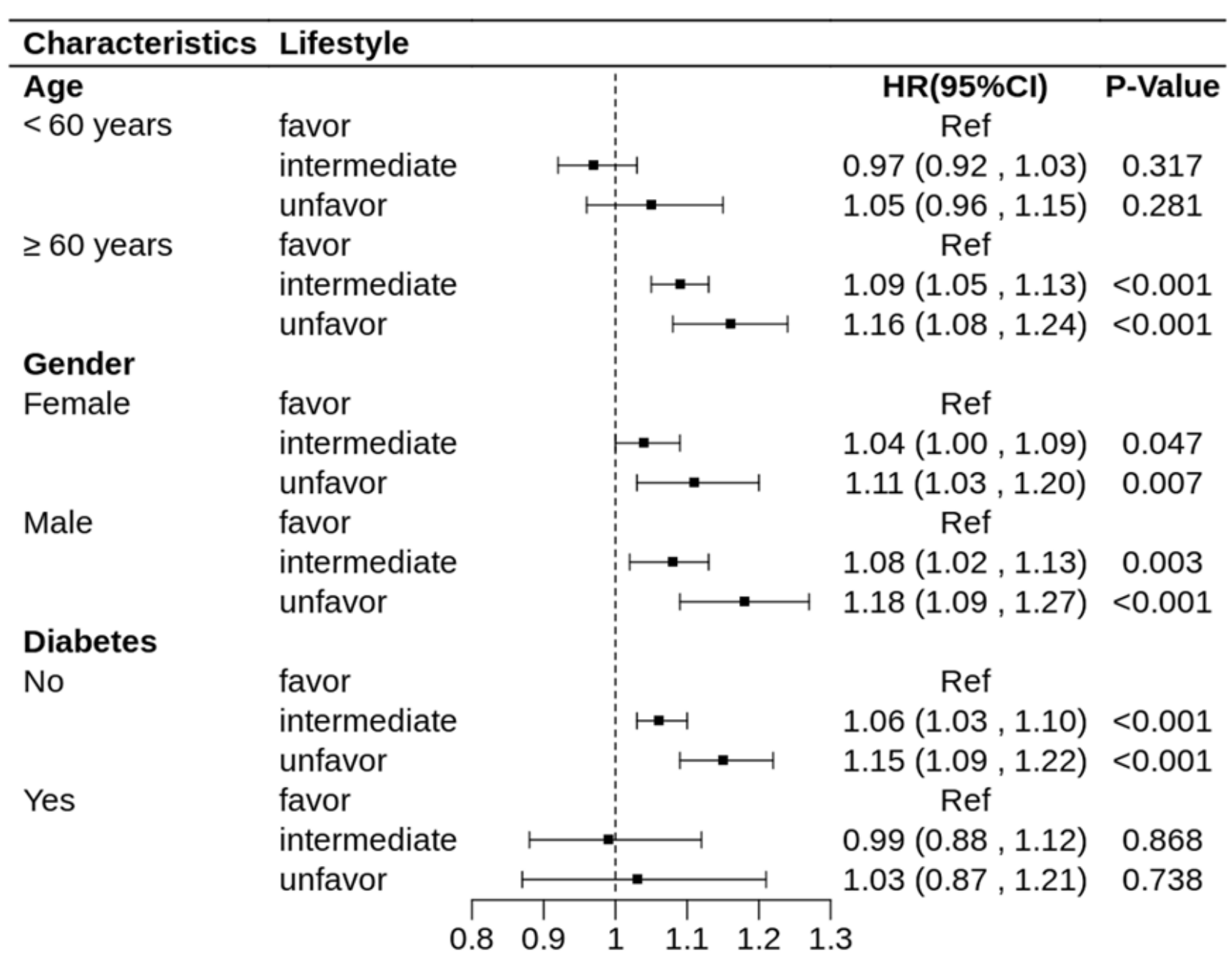

Supplementary Figure 2. Subgroup analysis according to age, gender and diabetes.

© 2025. Qi J et al. Published online at http://www.aginganddisease.org/EN/10.14336/AD.2025.0522

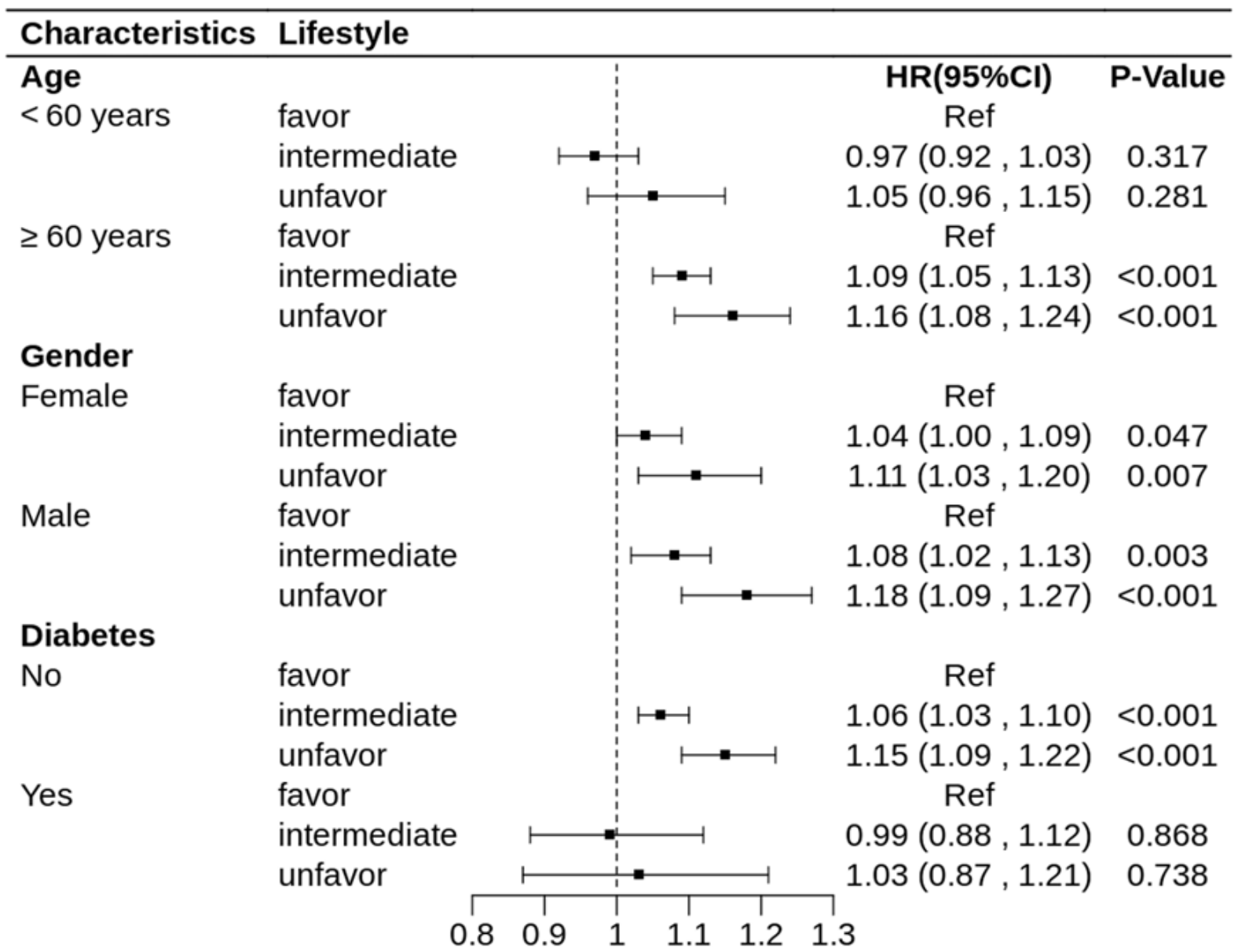

Supplementary Figure 2. Subgroup analysis according to age, gender and diabetes. Adjusted by age, gender, ethnicity, Townsend deprivation index (TDI), income, education, diabetes, hypertension. Abbreviations: HR: hazard ratio; CI, confidence interval.

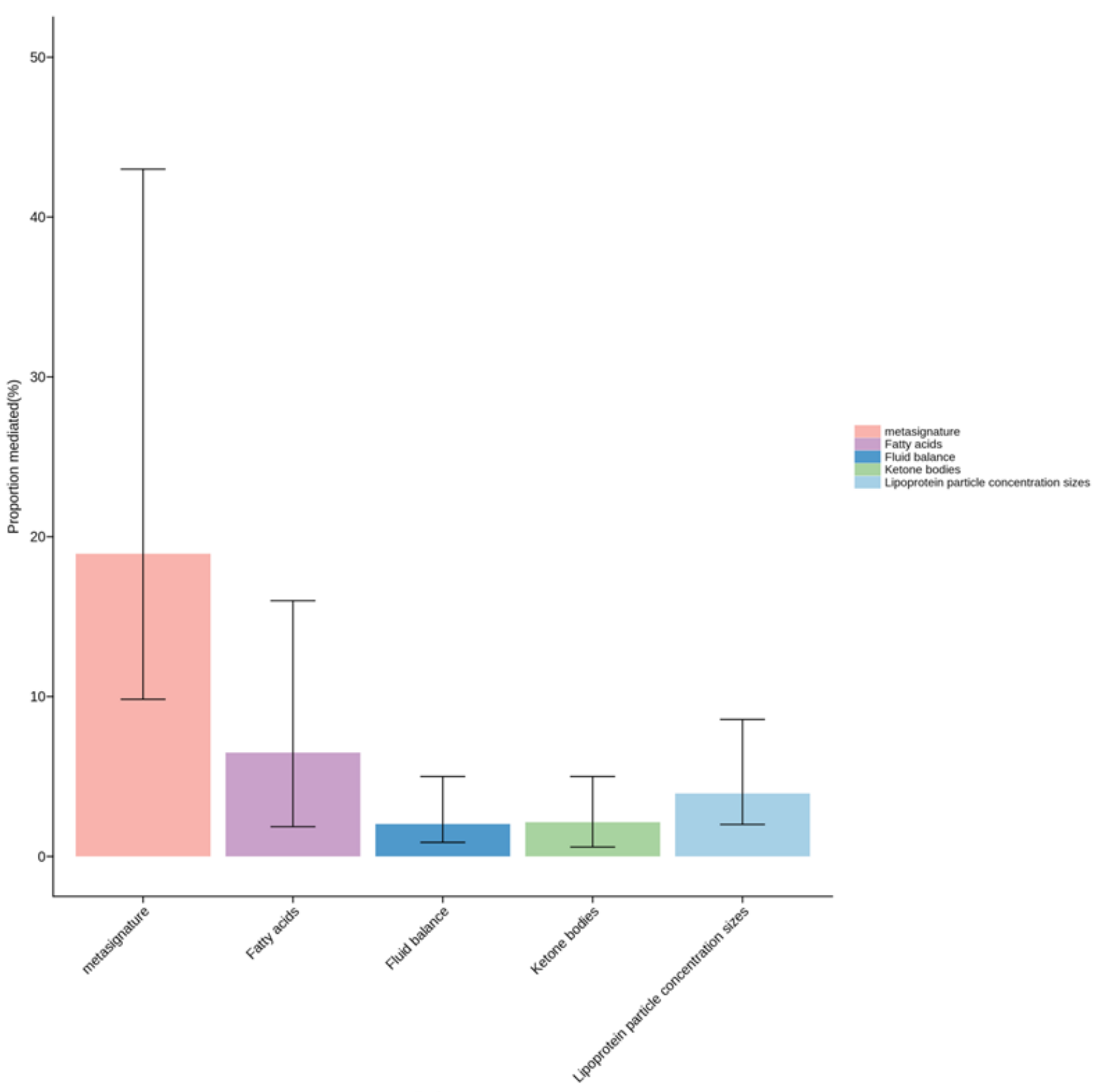

Supplementary Figure 3. Sensitivity analysis: Mediation proportions of metabolic pathway in the association between unhealthy lifestyle and the risk of cataract excluded participants who developed the cataract within 2 years.

© 2025. Qi J et al. Published online at http://www.aginganddisease.org/EN/10.14336/AD.2025.0522

SUPPLEMENTARY DATA

Supplementary Table 1. Exposures and corresponding data-fields in the UK Biobank

Unhealthy Lifestyles	Data-fields in the UK Biobank
Smoking	Smoking status（20116),<br>Light smokers, at least 100 smokes in lifetime（2644）
Drinking	Alcohol intake frequency (1558),<br>Average monthly red wine intake (4407),<br>Average monthly champagne plus white wine intake (4418),<br>Average monthly beer plus cider intake (4429),<br>Average monthly spirits intake (4440),<br>Average monthly fortified wine intake (4451),<br>Average monthly intake of other alcoholic drinks alcohol (4462),<br>Average weekly red wine intake (1568),<br>Average weekly champagne plus white wine intake (1578),<br>Average weekly spirits intake (1598),<br>Average weekly fortified wine intake (1608),<br>Average weekly intake of other alcoholic drinks (5364)
Physical Inactivity	Duration of vigorous activity (914),<br>Duration of moderate activity (894),<br>Time spent doing moderate physical activity (884),<br>Time spent doing vigorous physical activity (904)
Unhealthy BMI	BMI (21001)
Unhealthy Diet	Beef intake (1369),<br>Lamb/mutton intake (1379),<br>Pork intake (1389),<br>Processed meat intake (1349),<br>Oily fish intake (1329),<br>Non-oily fish intake (1339),<br>Fresh fruit intake (1309),<br>Dried fruit intake (1319),<br>Salad/raw vegetable intake (1299),<br>Cooked vegetable intake (1289),<br>Bread intake (1438),<br>Cereal intake (1458)

© 2025. Qi J et al. Published online at http://www.aginganddisease.org/EN/10.14336/AD.2025.0522

SUPPLEMENTARY DATA

Supplementary Table 2. Subgroup analysis: Associations of metabolic signatures with the risk of cataract

Characteristics	Metabolic signature[a]	
	HR (95% CI)	P-Value
**Age**		
< 60 years (n = 117,232)	1.23 (1.04-1.46)	0.017
≥ 60 years (n = 82,183)	1.29 (1.14-1.46)	<0.001
**Gender**		
Female (n = 103,054)	1.37 (1.18-1.58)	<0.001
Male (n = 96,361)	1.24 (1.06-1.44)	0.006
**Diabetes**		
No (n = 189,732)	1.29 (1.16-1.44)	<0.001
Yes (n = 9,683)	1.33 (0.96-1.84)	0.088

[a] Adjusted by age, gender, ethnicity, Townsend deprivation index (TDI), income, education, diabetes, hypertension.
Abbreviations: HR: hazard ratio; CI, confidence interval.

Supplementary Table 3. Subgroup analysis: proportions of mediated effects of metabolic signature on the associations of unhealthy lifestyle and cataract risk

Characteristics	Mediation proportion[a]（%）	
	Mediation proportion (95% CI)	P-Value
**Age**		
< 60 years (n = 117,232)	-34.56 (-315.11-494.36)	0.24
≥ 60 years (n = 82,183)	11.03 (2.63-22.62)	<0.001
**Gender**		
Female (n = 103,054)	28.87 (-24.27-134.94)	0.06
Male (n = 96,361)	10.77 (0.90-37.30)	0.04
**Diabetes**		
No (n = 189,732)	16.86 (5.80-32.35)	<0.001
Yes (n = 9,683)	-142.35 (-463.76-319.94)	0.90

[a] Adjusted by age, gender, ethnicity, Townsend deprivation index (TDI), income, education, diabetes, hypertension.
Abbreviations: HR: hazard ratio; CI, confidence interval.

Supplementary Table 4. Sensitivity analysis: Associations of unhealthy lifestyle score and metabolic signature with the risk of cataract (n = 197,968).

Group		Crude Model[a]		Model 1[b]		Model 2[c]	
		HR (95%CI)	P-Value	HR (95%CI)	P-Value	HR (95%CI)	P-Value
Lifestyle	Favor	Ref		Ref		Ref	
	Intermediate	1.14 (1.11-1.18)	< 0.001	1.08 (1.05-1.12)	< 0.001	1.06 (1.03-1.10)	< 0.001
	Unfavor	1.24 (1.17-1.30)	< 0.001	1.21 (1.15-1.28)	< 0.001	1.15 (1.09-1.22)	< 0.001
Metabolic signature		2.05 (1.86-2.26)	< 0.001	1.74 (1.57-1.93)	< 0.001	1.35 (1.21-1.50)	< 0.001

[a] Unadjusted (crude) model.
[b] Adjusted by age and gender.
[c] Adjusted by age, gender, ethnicity, Townsend deprivation index (TDI), income, education, diabetes, hypertension.
Abbreviations: HR: hazard ratio; CI, confidence interval.

© 2025. Qi J et al. Published online at http://www.aginganddisease.org/EN/10.14336/AD.2025.0522
